# Src Family Kinase Inhibitors Block Translation of Alphavirus Subgenomic mRNAs

**DOI:** 10.1128/AAC.02325-18

**Published:** 2019-03-27

**Authors:** Rebecca Broeckel, Sanjay Sarkar, Nicholas A. May, Jennifer Totonchy, Craig N. Kreklywich, Patricia Smith, Lee Graves, Victor R. DeFilippis, Mark T. Heise, Thomas E. Morrison, Nathaniel Moorman, Daniel N. Streblow

**Affiliations:** a Vaccine and Gene Therapy Institute, Oregon Health and Science University, Beaverton, Oregon, USA; b Division of Pathobiology and Immunology, Oregon National Primate Research Center, Beaverton, Oregon, USA; c Department of Genetics, The University of North Carolina at Chapel Hill, Chapel Hill, North Carolina, USA; d Department of Pharmacology, The University of North Carolina at Chapel Hill, Chapel Hill, North Carolina, USA; e Department of Microbiology and Immunology, The University of North Carolina at Chapel Hill, Chapel Hill, North Carolina, USA; f Department of Immunology and Microbiology, University of Colorado School of Medicine, Aurora, Colorado, USA

**Keywords:** chikungunya virus, alphavirus, antiviral agents

## Abstract

Alphaviruses are arthropod-transmitted RNA viruses that can cause arthralgia, myalgia, and encephalitis in humans. Since the role of cellular kinases in alphavirus replication is unknown, we profiled kinetic changes in host kinase abundance and phosphorylation following chikungunya virus (CHIKV) infection of fibroblasts. Based upon the results of this study, we treated CHIKV-infected cells with kinase inhibitors targeting the Src family kinase (SFK)–phosphatidylinositol 3-kinase (PI3K)–AKT–mTORC signaling pathways. Treatment of cells with SFK inhibitors blocked the replication of CHIKV as well as multiple other alphaviruses, including Mayaro virus, O’nyong-nyong virus, Ross River virus, and Venezuelan equine encephalitis virus. Dissecting the effect of SFK inhibition on alphavirus replication, we found that viral structural protein levels were significantly reduced, but synthesis of viral genomic and subgenomic RNAs was unaffected. By measuring the association of viral RNA with polyribosomes, we found that the SFK inhibitor dasatinib blocks alphavirus subgenomic RNA translation. Our results demonstrate a role for SFK signaling in alphavirus subgenomic RNA translation and replication. Targeting host factors involved in alphavirus replication represents an innovative, perhaps paradigm-shifting, strategy for exploring the replication of CHIKV and other alphaviruses while promoting antiviral therapeutic development.

## Abstract (precis)

Alphaviruses are arthropod-transmitted RNA viruses that can cause arthralgia, myalgia, and encephalitis in humans. Since the role of cellular kinases in alphavirus replication is unknown, we profiled kinetic changes in host kinase abundance and phosphorylation following chikungunya virus (CHIKV) infection of fibroblasts.

## INTRODUCTION

Alphaviruses are positive-sense, single-stranded RNA viruses in the *Togaviridae* family, and many of them are transmitted via a bite by infected mosquitoes. Some alphaviruses cause acute febrile illness and arthritic disease in humans, including O’nyong-nyong virus (ONNV), Mayaro virus (MAYV), Sindbis virus (SINV), and chikungunya virus (CHIKV) (1–3). The joint and muscle pain caused by CHIKV can be severe (4, 5), and the arthritic pain may last for several months to up to years after acute symptoms resolve (6, 7). Alphaviruses cause widespread outbreaks in areas of serologically naive individuals. In 2013, a CHIKV outbreak was initiated in the Caribbean (8), infecting an estimated 1.9 million people throughout Central and South America (http://www.paho.org/hq/index.php?option=com_topics&view=readall&cid=5927&Itemid=40931%3C=en). Other alphaviruses, such as Venezuelan equine encephalitis virus (VEEV), cause acute febrile illness and neurological disease, including fatal encephalitis (9, 10). In 1995, a VEEV epidemic in Colombia and Venezuela resulted in 75,000 to 100,000 human cases, with a small proportion of those cases developing encephalitis (11, 12). Treatment of all alphavirus infections, including CHIKV and VEEV, is currently limited to supportive care. New knowledge of the cellular requirements for alphavirus replication should facilitate the development of effective antiviral therapies to treat individuals suffering from alphavirus infections.

Alphavirus virions are internalized into host cells by clathrin-mediated endocytosis (13, 14), and the glycoproteins initiate viral fusion in endosomal compartments, releasing the viral genome into the cytoplasm. The nonstructural protein precursors P123 and P1234 are translated from the incoming capped and polyadenylated genomic RNA (15, 16). The nonstructural proteins form the replication complex in spherules at the plasma membrane (17–22). Minus-stranded RNA is synthesized by P123 and cleaved nsP4, while full-length positive-sense viral genomic RNA and the subgenomic mRNA (sgmRNA) are synthesized by cleaved nonstructural protein P1234 (23, 24). The translated subgenomic polyprotein is cleaved by virus- and host-mediated proteolytic activity into the mature structural proteins capsid (C), E3, E2, 6K/TF, and E1 (25–27). Translation of the sgmRNA is efficient, even though CHIKV infection induces inactivating phosphorylation of the α subunit of eukaryotic translation initiation factor 2 (eIF2α) (28). In addition, translation of the SINV sgmRNA does not appear to require eIF4G (29) or the noncanonical translation initiation factors eIF2D and eIF2A (29, 30). Translation of SINV sgmRNA is facilitated by a GC-rich structural element called the downstream loop (DLP), which allows for translation in the absence of eIF2 (28, 31, 32). However, CHIKV and VEEV have no predicted DLP structure (31), but rather, they may have an alternative mechanism for efficient sgmRNA translation.

Viruses utilize and modulate cellular signaling pathways to promote an intracellular environment suitable for viral replication (33, 34) by influencing metabolism, growth, differentiation, transcription, translation, and cytoskeletal rearrangement (35, 36). One key group of kinases involved in controlling some of these cellular processes is the Src family kinases (SFKs) (37–41). Nine Src family kinase members have been described: Src, Fyn, Yes, Blk, Fgr, Hck, Lck, Yrk, and Lyn. SFKs are myristoylated and sometimes palmitoylated (42) membrane-associated proteins that mediate signal transduction of many receptors, including G-protein-coupled receptors (43), receptor tyrosine kinases (44), and integrins (45). Many viruses encode proteins that directly interact with and modulate SFK activity (46–49), and therefore, SFK inhibitors have been employed to study the role of SFKs in virus replication. For example, SFK inhibitors block West Nile virus (WNV) envelope protein trafficking and maturation (50). The SFK inhibitor dasatinib inhibits the replication of dengue virus (DENV) (51, 52), human immunodeficiency virus (HIV) (53), and hepatitis C virus (HCV) (54). Dasatinib affected multiple stages of DENV replication by reducing DENV RNA accumulation (52) and preventing virion assembly in the endoplasmic reticulum (ER) (51). Dasatinib suppressed HIV replication by blocking reverse transcription and integration (53, 55). In addition, dasatinib was shown to block HCV fusion during entry by inhibiting ephrin 2A (Eph2A) kinase-mediated signaling necessary for CD81-CLDN1 cofactor complex formation (54, 56). Therefore, a precedent exists for utilizing SFK inhibitors to identify steps in viral replication that require Src-related kinase activity.

Identifying the specific cellular kinases and signaling pathways that promote alphavirus replication could aid in the development of antialphavirus compounds. In order to identify kinase pathways important for alphavirus replication, we profiled changes in kinase abundance and/or activity following CHIKV infection. We then tested the antiviral activities of inhibitors of cellular pathways that were altered by infection, including the SFK–phosphatidylinositol 3-kinase (PI3K)–Akt–mTOR pathway. SFK inhibitors (PP2 and dasatinib) and mTORC1/2 inhibitors (PP242 and Torin 1) block alphavirus replication in human fibroblasts. While dasatinib and Torin 1 had minimal effects on viral RNA (vRNA) synthesis, each treatment blocked structural protein accumulation during CHIKV and VEEV replication. Furthermore, dasatinib decreased the amount of CHIKV RNA associated with polysomes, indicating that CHIKV relies on SFKs and mTORC1/2 for structural protein synthesis. Our novel findings provide insight into molecular processes that comprise the CHIKV replication cycle, including the requirement of SFK activity for alphavirus replication.

## RESULTS

### Kinome pathway analysis of CHIKV-infected fibroblasts.

**FIG 1:**
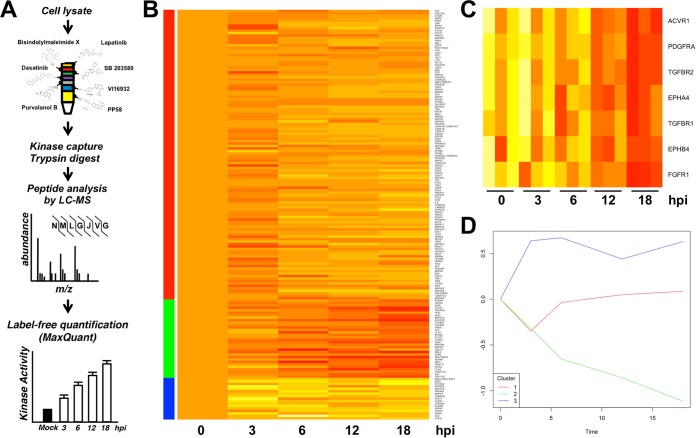
MIBS profiling identifes changes to the cellular kinome induced by CHIKV infeciton. Serum-starved NHDFs were infected with CHIKV (MOI = 5 PFU/cell); harvested at 3, 6, 12, or 18 hpi; and analyzed by MIB-MS kinome profiling. (A) Schematic showing the procedural workflow. (B) Heat map showing average relative kinase changes (*n* = 3) compared to uninfected cells (0 hpi). Bars to the left of the heat map denote kinase clusters. (C) Examples of kinases significantly downregulated after CHIKV infection. (D) Graph showing average changes in activity/expression of kinases in clusters 1, 2, and 3.

To identify kinase pathways that are kinetically altered following CHIKV infection, we utilized multiplexed kinase inhibitor beads coupled with quantitative mass spectrometry (MIB-MS) kinome profiling to quantify changes to the cellular kinome over a time course of infection (57, 58). Kinome profiling uses broad-spectrum kinase inhibitors coupled to a matrix as an affinity reagent to capture kinases from cell lysates. The identity and abundance of the recovered kinases are then determined by quantitative mass spectrometry. Changes in the amounts of kinase recovered under different conditions reflect changes in the expression or activity of the kinase. As depicted in Fig. 1A, serum-starved normal human dermal fibroblasts (NHDFs) were infected with CHIKV_181/25_ (multiplicity of infection [MOI] = 3 PFU/cell), and lysates were collected at 4, 8, 12, and 24 h postinfection (hpi) for kinome profiling. Infection induced multiple changes to the kinome (Fig. 1B). Examples of kinases significantly altered after infection are shown in Fig. 1C. These kinases include the transforming growth factor β (TGF-β) receptor subunits and members of the ephrin family. Other kinases showed consistent trends but failed to achieve significance due to variability. A comparative analysis of kinome changes revealed that kinases clustered into three distinct groups by k-means clustering. Kinases in cluster 1 (118 kinases) remained stable across the course of infection. Levels of kinases in cluster 2 (32 kinases) were decreased by infection, and those of kinases in cluster 3 (17 kinases) were increased (Fig. 1D; see also Table S2 in the supplemental material). These data suggest that CHIKV infection significantly alters the expression and/or activity of the cellular kinome.

Changes in kinase recovery measured by kinome profiling can arise from changes in either kinase expression or kinase activity due to phosphorylation. To more specifically identify changes in phosphorylation, CHIKV-infected cell lysates were collected at 0, 1, 2, 4, 6, 8, and 24 hpi and analyzed via PathScan receptor tyrosine kinase (RTK) signaling antibody arrays spotted with RTK phosphoantibodies (Fig. S1). Increased phosphorylation of Akt, platelet-derived growth factor receptor (PDGFR), and extracellular signal-regulated kinase 1/2 (ERK1/2) was detected at early times postinfection (1 to 2 hpi). Phosphorylation of the ephrins (EphA1, EphB4, and EphB1), Axl, Src, Lck, and IRS-1 occurred after 2 hpi. These findings confirm the kinome profiling results and support the finding that the SFK-PI3K-Akt-mTOR signaling pathway is activated following CHIKV infection.

### The Src family kinase inhibitor dasatinib blocks CHIKV replication and reduces CHIKV-induced cell death.

**FIG 2:**
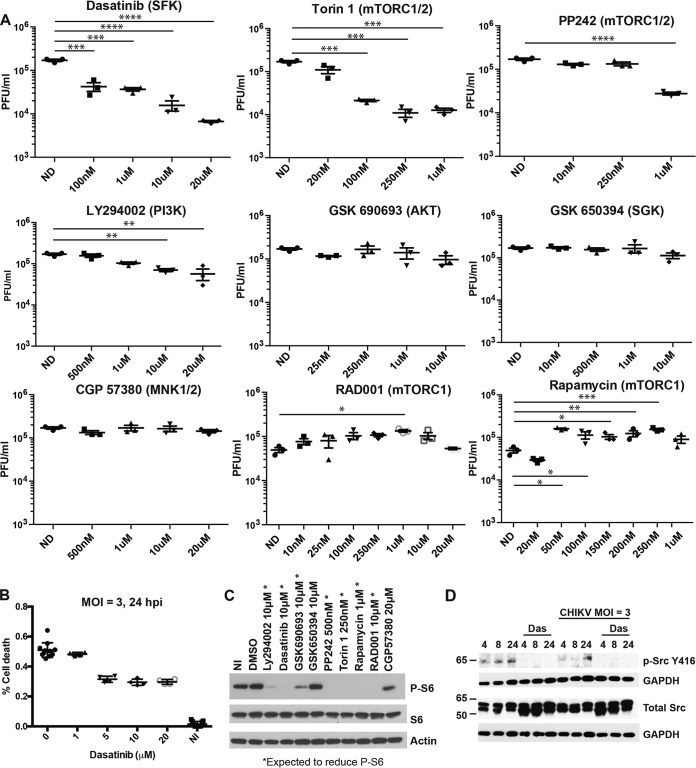
Dasatinib (Das), Torin 1, and PP242 block CHIKV replication in human fibroblasts. (A) NHDFs were pretreated for 2 h with drug and infected with CHIKV_SL15649_ (MOI = 1 PFU/cell). Cells were washed twice with PBS at 2 hpi, and inhibitor-containing medium was added to the cells. Supernatants from infected cells were collected at 20 hpi, and titers were determined on Vero cells by a limiting-dilution plaque assay. ND, not done. (B) NHDFs were infected with CHIKV (MOI = 3 PFU/cell) and treated with dasatinib at 2 hpi. At 24 hpi, cells were analyzed for viability using the CellTiter-Glo viability assay kit. NI, not infected; DMSO, dimethyl sulfoxide. (C) NHDFs were treated as described above for panel A, and cell lysates were analyzed by Western blotting for p-S6, S6, and actin. (D) NHDFs were infected with CHIKV_181/25_ (MOI = 3 PFU/cell) or left uninfected. At 2 hpi, cells were washed twice with PBS, and medium was replaced, with or without drug. Cell lysates were collected at the indicated times. Western blot membranes were probed with anti-p-SFK (Tyr416) and anti-GAPDH antibodies. Statistics were performed on log-transformed data, multiple comparisons were performed using Dunn’s multiple-comparison test, and multiplicity-adjusted *P* values are reported (*n* = 3) (*, *P* < 0.05; **, *P* < 0.005; ***, *P* < 0.0005; ****, *P* < 0.00005).

Both of the kinome profiling experiments showed that phosphorylated Src (p-Src) and p-Akt levels change over time during CHIKV infection. To investigate the functional importance of the SFK-PI3K-Akt-mTOR signaling pathway, chemical inhibitors of this pathway were tested for their ability to block CHIKV replication. To ensure that the inhibitors were used at concentrations that were not cytotoxic, cell viability assays were performed in uninfected NHDFs 24 h after drug treatment (Fig. S2). Inhibitor activity was verified in NHDFs by Western blotting for the presence of phosphorylated ribosomal protein S6 (p-rpS6) (Fig. 2C), a common marker of mTOR activation (59, 60). To assess the effects of the SFK-PI3K inhibitors on CHIKV replication, NHDFs were pretreated for 2 h with the inhibitor and infected with CHIKV_SL15649_ (MOI = 1 PFU/cell) in the presence of drug. At 2 hpi, the inoculum was removed, cells were washed with phosphate-buffered saline (PBS), and new drug-containing medium was added. Supernatants were collected at 20 hpi, and virus titers were determined by serial-dilution plaque assays on Vero cells. Dasatinib and Torin 1 treatments reduced yields of infectious virus over 10-fold (Fig. 2A) and reduced CHIKV-mediated cytotoxicity (Fig. 2B). PP242 and LY294002 treatments mildly reduced CHIKV yields. In contrast, treatment with GSK 690693, GSK 650394, and CGP 57380 had no significant effect on CHIKV replication. Treatment with RAD001 and rapamycin slightly increased CHIKV replication in NHDFs (Fig. 2A), which is consistent with data from previous reports (61–63).

Of the inhibitors tested, dasatinib treatment resulted in the strongest inhibition of CHIKV replication. Dasatinib is a potent inhibitor of SFKs (64, 65). As confirmation of this activity in NHDFs, the activating phosphorylation of Src at Tyr416 was analyzed in cell lysates at 4, 8, and 24 hpi (66). In the absence of dasatinib, there was an increase of Src Tyr416 phosphorylation in CHIKV-infected cells compared with uninfected cells. Dasatinib reduced Src phosphorylation in both infected and uninfected cells (Fig. 2D).

**FIG 3:**
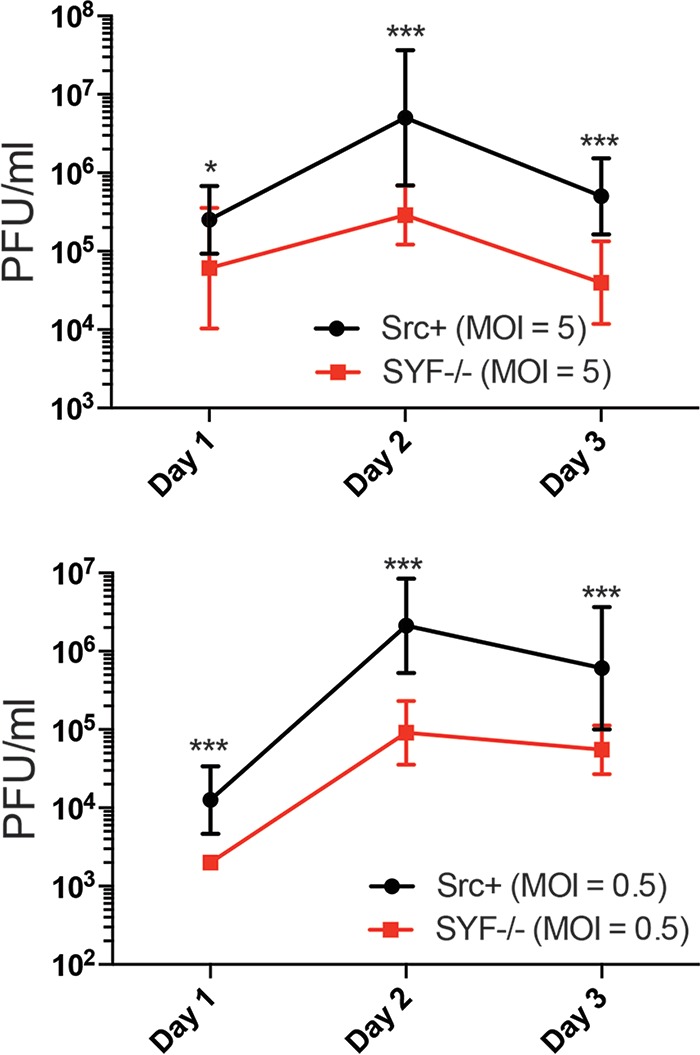
CHIKV replication is suppressed in the absence of Src. SYF^−/−^ cells (MEF knockout [K/O] of Src, Yes, and Fyn) and Src^+^ cells (SYF^−/−^ cells stably transfected with Src) were infected at an MOI of 5 PFU/cell (top) or an MOI of 0.5 PFU/cell (bottom); supernatants were collected at 1, 2, and 3 days postinfection; and titers were determined by a plaque assay. Statistics were performed on log-transformed data and analyzed by Sidak’s multiple-comparison test, and multiplicity-adjusted *P* values are reported (*n* = 3) (*, *P* < 0.05; ***, *P* < 0.0005).

Dasatinib has many cellular targets, including Src, Lck, Lyn, Yes, Fyn, ephrin receptors, and Bcr/Abl (67). To confirm the importance of Src in CHIKV replication, CHIKV growth was measured in SYF^−/−^ mouse embryonic fibroblasts (MEFs) (lacking Src, Yes, and Fyn) and Src^+^ SYF^−/−^ MEFs, in which Src was stably reintroduced (68). At each time point evaluated, yields of infectious CHIKV from Src^+^ SYF^−/−^ MEFs were consistently higher than those from SYF^−/−^ MEFs at both high and low MOIs (Fig. 3). These data are consistent with the hypothesis that Src is required for efficient CHIKV replication.

**FIG 4:**
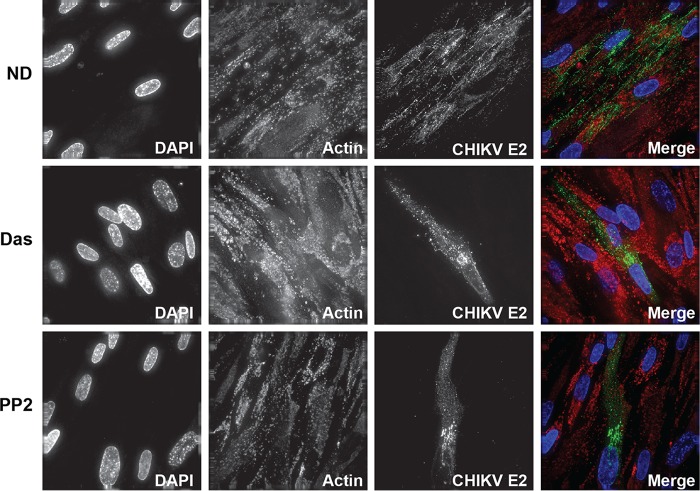
CHIKV replication and spread are limited by dasatinib and PP2. NHDFs were infected with CHIKV_181/25_ (MOI = 1 PFU/cell) and treated with dasatinib or PP2 at 2 hpi. At 24 hpi, cells were fixed, stained for virus-specific envelope glycoproteins (green), and counterstained with DAPI (blue) and phalloidin (red) to highlight host DNA and actin filaments.

To validate the inhibition of CHIKV replication, immunofluorescence staining was performed on CHIKV-infected cells treated with dasatinib or another SFK inhibitor, PP2. Cells were fixed at 24 hpi and stained with antibodies directed against CHIKV E2 and actin as well as the DNA stain DAPI (4′,6-diamidino-2-phenylindole). Foci of CHIKV-infected cells were observed in nontreated cells, whereas dasatinib and PP2 treatments resulted in the presence of single infected cells (Fig. 4). Similar findings were observed in VEEV_TC83_-infected cells, where SFK inhibitors blocked the ability of the virus to spread through the culture (Fig. S3). Combined, these data indicate that SFK inhibitors block virus spread by reducing infectious virus production from infected cells.

### Dasatinib and Torin 1 block alphavirus infection in a type I interferon-independent manner.

CHIKV infection activates interferon (IFN) regulatory factor 3 (IRF3), resulting in the transcription of type I interferon, which blocks viral replication (69). Src can promote the phosphorylation of TANK-binding kinase 1 (TBK1), which activates IRF3 and promotes type I IFN production (70). Therefore, SFK inhibitors have the potential to augment CHIKV replication via repression of type I IFN responses. To test whether dasatinib-mediated inhibition of CHIKV replication was dependent on IRF3, human fibroblasts deficient in IRF3 (THF-ISRE-ΔIRF3) (71) were infected with CHIKV_181/25_ or VEEV_TC83_ and treated at 0 hpi or 2 hpi with dasatinib. Titers in supernatants collected at 24 hpi were determined by a serial-dilution plaque assay, and the results showed that replication of CHIKV and VEEV was inhibited by dasatinib treatment in IRF3^−/−^ cells (Fig. S4A). Another component of the interferon response is the protein signal transducer and activator of transcription 1 (STAT1), which acts downstream of the type I interferon receptor and activates the transcription of interferon-stimulated genes. STAT1 knockout telomerized human fibroblasts (THFs) (THF-ISRE-ΔSTAT1) (71) were infected with CHIKV_181/25_ or VEEV_TC83_ and treated with dasatinib or Torin 1. Quantification of viral yields at 24 hpi revealed that CHIKV or VEEV maintained sensitivity to dasatinib in STAT1^−/−^ THFs (Fig. S4B). Together, these data indicate that the mechanism of dasatinib and Torin 1 inhibition of CHIKV replication is independent of their potential effects on IRF3- and STAT1-mediated innate immunity.

### Torin 1 and dasatinib differentially regulate autophagy.

Autophagy is an important downstream pathway of mTOR, which is induced when mTOR is inhibited (72). There are conflicting reports regarding whether CHIKV replication is facilitated or impaired by autophagy (61, 63, 73), but both Torin 1 and dasatinib block mTOR signaling. Therefore, we next determined whether Torin 1 and dasatinib treatments induced autophagy in NHDFs and whether this impacted alphavirus replication. CHIKV_181/25_-infected cells were treated with dasatinib, Torin 1, or 3-methyladenine (3-MA) (1 mM), an inhibitor of autophagy. At 7 hpi, cell lysates were harvested and evaluated by Western blotting to examine the proteolytic cleavage of LC3-I to LC3-II, a conventional indicator of the induction of autophagy (74). Torin 1 induced LC3-II accumulation, but dasatinib and 3-MA did not alter LC3 status compared with untreated infected cells (Fig. S5A). Blotting cellular lysates for E2 showed that while dasatinib and Torin 1 reduced E2 levels, 3-MA increased CHIKV E2 accumulation compared with the untreated control (Fig. S5B). Our data indicate that Torin 1 and dasatinib inhibit CHIKV replication via different mechanisms: Torin 1 may block virus through the induction of autophagy, while dasatinib may have an alternative mechanism.

### Dasatinib does not block CHIKV RNA replication.

**FIG 5:**
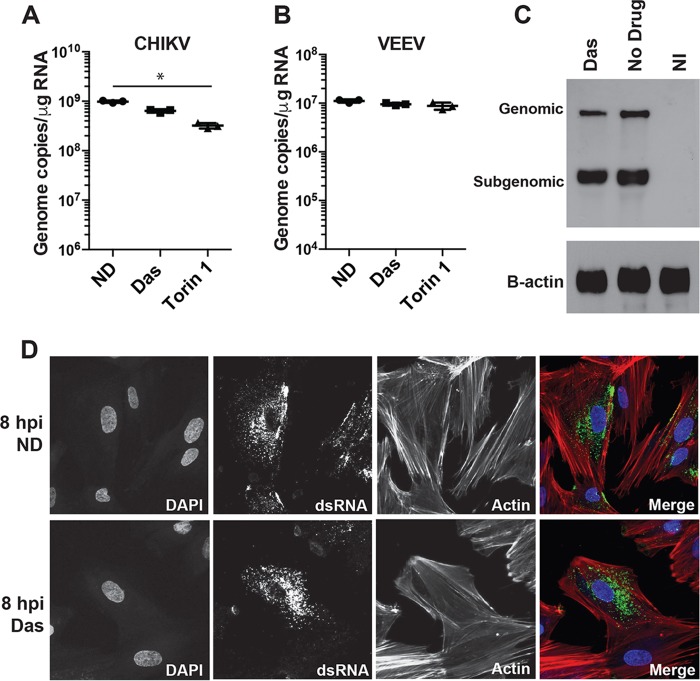
SFK inhibitors do not reduce CHIKV RNA synthesis or replication complex formation. (A and B) NHDFs were infected with CHIKV_181/25_ (A) or VEEV (B) (MOI = 3 PFU/cell). At 2 hpi, cells were washed twice with PBS, and cells were treated with 10 μM dasatinib. At 12 hpi, cells were washed extensively in PBS, and cells were lysed with TRIzol reagent for total RNA isolation. Relative levels of vRNA were analyzed by qRT-PCR using gene-specific primers and probes directed against CHIKV E1 and VEEV E2. Statistical analyses were performed on log-transformed data, data were analyzed by Dunn’s multiple-comparison test, and multiplicity-adjusted *P* values are reported (*n* = 3) (*, *P* < 0.05). (C) RNAs from uninfected and CHIKV_181/25_-infected cells (MOI = 3 PFU/cell) treated with and without dasatinib were analyzed by Northern blotting for genomic and subgenomic RNA levels at 12 hpi. (D) NHDFs were infected with CHIKV_181/25_ (MOI = 25 PFU/cell) and treated with dasatinib at 2 hpi or left untreated (ND). At 8 hpi, cells were fixed and stained for dsRNA (J2) (green), actin (phalloidin) (red), and DNA (DAPI) (blue).

To further identify the step in the virus replication cycle sensitive to SFK inhibition, viral RNA (vRNA) accumulation was monitored in dasatinib- or Torin 1-treated NHDFs infected with CHIKV_181/25_ or VEEV_TC83_ (MOI = 3 PFU/cell). Viral RNA levels were measured by reverse transcription-quantitative PCR (qRT-PCR) at 12 hpi. Dasatinib and Torin 1 modestly reduced CHIKV RNA levels (Fig. 5A) but had no discernible effect on VEEV RNA accumulation (Fig. 5B). To validate and further explore the lack of effects of dasatinib on vRNA production, we performed a Northern blot analysis on RNA isolated from treated NHDFs infected with CHIKV (MOI = 3 PFU/cell). A double-stranded DNA (dsDNA) digoxigenin (DIG)-labeled probe recognizing E2-6K-E1 was generated to distinguish between genomic vRNA and subgenomic vRNA. Dasatinib treatment minimally reduced the levels of genomic and subgenomic CHIKV RNAs to 79% and 98% of the viral RNA levels of the untreated control, respectively (Fig. 5C). qRT-PCR analysis confirmed that the ratios of CHIKV nsP2 and E2 RNA levels were unaffected by dasatinib treatment (Fig. S6A and S6B). Combined, these data indicate that CHIKV RNA amplification in the presence of dasatinib is largely unaffected, and the minimal effects on RNA levels could be attributed to the abrogation of subsequent rounds of replication due to the block in the production of infectious virus.

Next, we determined whether dasatinib disrupts CHIKV replication complex formation. At 8 hpi, replication complexes were identified by immunofluorescence staining with an antibody specific for dsRNA (21, 75). The dsRNA-positive complexes appeared indistinguishable between dasatinib-treated cells and untreated cells (Fig. 5D). Additionally, there were no visual differences in the amounts of dsRNA staining at 8 hpi (Fig. S6C), which is consistent with our data demonstrating that dasatinib affects CHIKV replication downstream of vRNA synthesis.

### Dasatinib blocks CHIKV and VEEV structural protein production.

**FIG 6:**
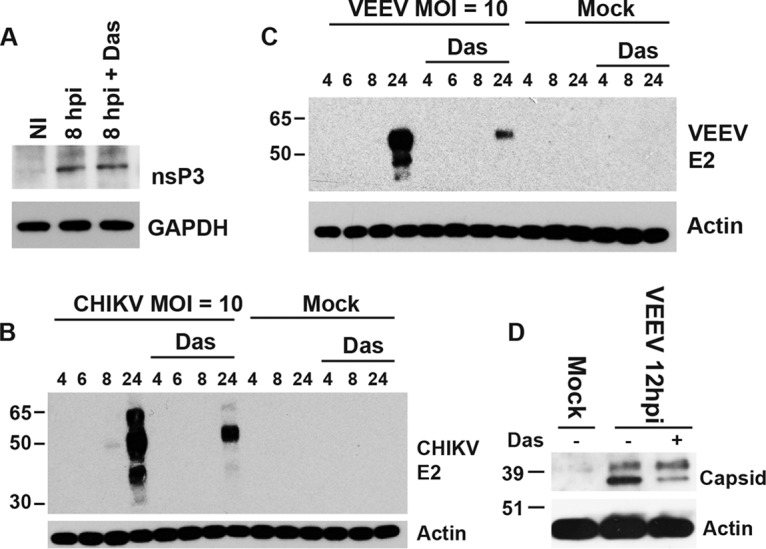
Dasatinib blocks structural protein accumulation. (A) NHDFs were infected with CHIKV_181/25_ (MOI = 10 PFU/cell) and treated with dasatinib at 2 hpi, and cell lysates were analyzed for nsP3 protein levels at 8 hpi by Western blotting. (B and C) NHDFs were infected with CHIKV_181/25_ (B) or VEEV_TC83_ (C) (MOI = 10 PFU/cell), and E2 protein levels were analyzed at the indicated times by Western blotting. (D) For comparison with another structural protein, NDHFs were infected with VEEV_TC83_ (MOI = 1 PFU/cell), treated with dasatinib at 2 hpi, and analyzed for VEEV capsid levels by Western blotting.

We hypothesized that dasatinib does not influence the synthesis of viral nonstructural proteins since viral RNA levels and replication complex formation appeared normal in treated cells. To directly test this hypothesis, Western blot analysis for nsP3 was performed on lysates from treated CHIKV-infected cells at 8 hpi. We observed no difference in nsP3 levels with dasatinib treatment (Fig. 6A) in the presence of a strong reduction in E2 protein accumulation (Fig. 6B). Similarly, levels of VEEV E2 glycoprotein (Fig. 6C) and capsid (Fig. 6D) were also dramatically reduced in the presence of the dasatinib. Together, these data suggest that dasatinib blocks the synthesis of alphaviral structural proteins but has minimal effects on the production of vRNA or nonstructural proteins.

**FIG 7:**
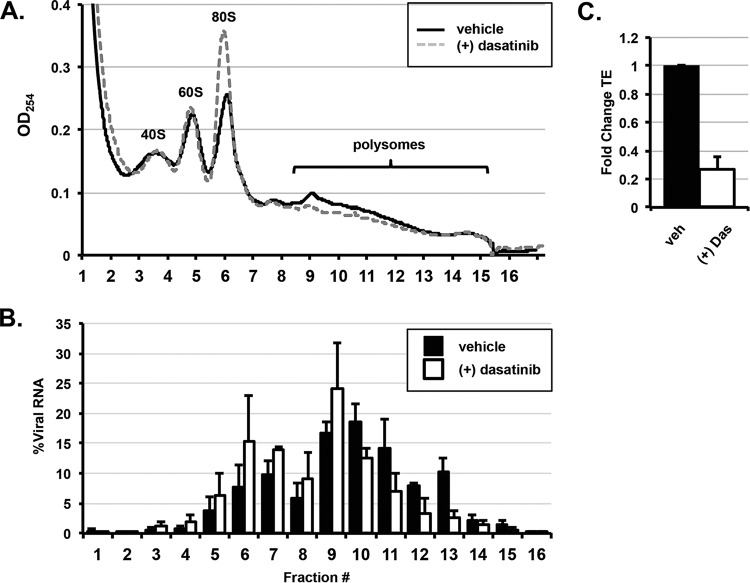
Dasatinib decreases the translation efficiency of CHIKV RNA. (A) NHDFs infected with CHIKV (MOI = 3 PFU/cell) were untreated or treated with 10 μM dasatinib. Cytoplasmic lysates of infected cells were collected at 12 hpi and resolved through 10 to 50% linear sucrose gradients. The presence of ribosomal subunits (40S and 60S), monosomes (80S), and polysomes was monitored by continuous measurement of the absorbance (OD_254_) during fractionation. (B) Total RNA was prepared from each fraction, and the percentage of CHIKV RNA per fraction was determined by qRT-PCR as a percentage of the total cytoplasmic viral RNA. (C) The translation efficiency (TE) of CHIKV RNA under each condition was determined by qRT-PCR. The fold change in translation efficiency for CHIKV RNA in the presence of dasatinib is reported. The translation efficiency of the vehicle-treated control cells is set to 1 (*n* = 2).

Next, we sought to determine the mechanism by which dasatinib blocks CHIKV structural protein accumulation. Ribosomal profiling was performed on lysates from CHIKV-infected, inhibitor-treated NHDFs at 12 hpi. Lysates were fractionated by centrifugation over a linear sucrose gradient, and 17 fractions were collected and analyzed for the optical density at 264 nm (OD_264_) as well as for the level of vRNA by qRT-PCR. Absorbance readings of the fractions revealed that dasatinib caused an increase of the absorbance in the 80S monosomal peak and a decrease in the absorbance in polysome fractions (fractions 9 to 15) (Fig. 7A). In dasatinib-treated cells, the vRNA was shifted into fractions containing monosomes (Fig. 7B). The translation efficiency (ratio of viral RNA levels in the polysome fractions to those in the fractions containing ribosomal subunits and monosomes) was reduced by approximately 80% in dasatinib-treated samples (Fig. 7C). These results indicate that dasatinib treatment disrupted CHIKV structural protein synthesis by preventing the translation machinery from associating with viral sgmRNA.

### Dasatinib does not inhibit alphavirus-mediated host translational shutoff.

**FIG 8:**
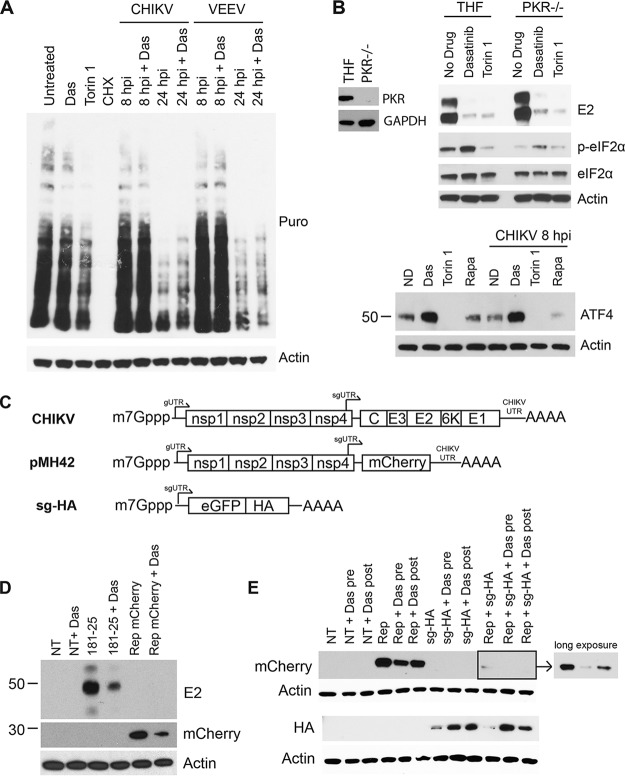
Dasatinib blocks translation of sgmRNAs from replicon-containing cells. (A) NHDFs were infected with CHIKV or VEEV (MOI = 10 PFU/cell) or left uninfected. At 2 hpi, cells were treated with 10 μM dasatinib, 250 nM Torin 1, or 200 μg/ml cyloheximide (CHX), as indicated. At 8 hpi or 24 hpi, cells were pulsed with 10 μg/ml puromycin (Puro) for 15 min. Following treatment, puromycin-free medium was added to the cells for 1 h, and lysates were collected and analyzed by Western blotting for puromycin incorporation and actin. (B) PKR^−/−^ THFs or parental THFs were assessed for the presence of PKR and GAPDH by Western blotting (left). PKR^−/−^ THFs or parental THFs were infected with CHIKV_SL15649_ (MOI = 3 PFU/cell). At 8 hpi, lysates were analyzed by Western blotting for E2, p-eIF2α, eIF2α, and actin (right). NHDFs were uninfected or infected with CHIKV and treated with the inhibitor. At 8 hpi or 8 h posttreatment, cell lysates were collected and analyzed for ATF4 and actin by Western blotting (bottom). Rapa, rapamycin. (C) Schematic of *in vitro*-synthesized mRNAs. (D) Two micrograms of CHIKV_181/25_ mRNA or pMH42 mRNA was transfected into NHDFs and treated with 10 μM dasatinib at 45 min posttransfection. At 20 h posttransfection, cell lysates were analyzed for CHIKV E2, mCherry, or actin by Western blotting. (E) NHDFs were pretreated with 10 μM dasatinib for 30 min prior to transfection or posttreated 30 min after transfection, as indicated. mRNAs were transfected with 2 μg pMH42, sg-HA, or a combination of both mRNAs. Lysates were collected at 20 h posttransfection and analyzed by Western blotting for mCherry, HA, and actin.

The precise mechanism of translation of CHIKV and VEEV sgmRNAs during host transcription and translation shutoff is not known (76). Translation of the sgmRNA may not require many of the canonical translation initiation factors, as was previously documented for SINV (29, 30, 77). To determine whether dasatinib affects the virus-induced host translation shutoff process, we performed a puromycin pulse-chase assay in NHDFs infected with CHIKV or VEEV. Although dasatinib significantly decreased viral E2 accumulation, there was no effect of treatment on virally induced host translation shutoff at 24 hpi (Fig. 8A). Additionally, dasatinib did not affect puromycin incorporation into newly synthesized protein in uninfected cells, whereas cycloheximide (CHX) treatment abolished protein production, and Torin 1 reduced global protein production (Fig. 8A).

A marker for the shutoff of cellular translation during viral infection is the phosphorylation status of the alpha subunit of eukaryotic initiation factor 2 (eIF2α), a host initiation factor essential for the translation of capped mRNAs. Phosphorylation inactivates eIF2α, thereby preventing cap-dependent cellular translation. Translation of CHIKV sgmRNA likely does not require eIF2 because the sgmRNA is translated efficiently when eIF2α is phosphorylated (69). One of the kinases responsible for eIF2α phosphorylation during CHIKV replication is the cytoplasmic dsRNA sensor protein kinase R (PKR), which is activated by CHIKV infection (69). Therefore, we determined the effects of dasatinib and Torin 1 treatments on CHIKV replication in cells lacking PKR. Compared with the parental cell line, dasatinib and Torin 1 still reduced E2 levels in PKR^−/−^ THFs (Fig. 8B). Consistent with previously reported data, CHIKV infection induced eIF2α phosphorylation in a PKR-dependent manner. In addition, dasatinib treatment increased eIF2α phosphorylation in both the parent THF cell line and PKR^−/−^ THFs, suggesting that dasatinib induces eIF2α phosphorylation in a PKR-independent manner. Since dasatinib induces eIF2α phosphorylation, we tested whether activating transcription factor 4 (ATF4) levels were affected, as they are expected to increase when eIF2 is phosphorylated/inactivated (78–80). There was an increase in ATF4 levels following the addition of dasatinib, suggesting that dasatinib induces a stress response in the cell. Despite inducing eIF2α phosphorylation and increasing ATF4 levels, dasatinib did not have an effect on global protein synthesis, as shown by puromycin incorporation (Fig. 8A).

We next tested whether dasatinib could block translation from *in vitro*-transcribed and capped mRNAs transfected into NHDFs. mRNA was generated by *in vitro* transcription from the CHIKV_181/25_ cDNA clone or a CHIKV replicon (pMH42), where mCherry replaces the CHIKV structural genes (Fig. 8C). Transfection of these mRNAs into cells results in nsP production, replication complex formation, and sgmRNA translation, leading to mCherry (replicon derived) or the viral structural proteins (cDNA clone derived). NHDFs were treated with dasatinib, and the production of E2 or mCherry was quantified at 20 h posttransfection (hpt) by Western blotting. Levels of pE2 were reduced following dasatinib treatment in mRNA-transfected NHDFs (Fig. 8D), and mCherry expression was decreased in pMH42-transfected cells, confirming that dasatinib blocks the translation of the sgmRNA.

To determine whether dasatinib could block sgmRNA outside the context of the replicon, another mRNA was generated, which contained the CHIKV 5′ subgenomic untranslated region (sgUTR) followed by a hemagglutinin (HA)-tagged version of enhanced green fluorescent protein (eGFP) (sg-HA) (Fig. 8C). mRNAs derived from the pMH42 mCherry-expressing replicon, sg-HA, or a combination of both were transfected into NHDFs with and without dasatinib treatment. Consistent with our above-described data, levels of mCherry generated from the replicon were reduced after treatment with dasatinib (Fig. 8E). In contrast, samples transfected with sg-HA mRNA demonstrated increased HA levels in the presence of dasatinib. When both mRNAs were transfected together, mCherry levels were reduced relative to those with replicon transfection alone, but expression of mCherry was blocked in the presence of dasatinib, while HA levels were increased relative to those in untreated cells. These data demonstrate that dasatinib blocks the translation of sgmRNAs generated from replication-derived complexes but does not block translation of mRNAs outside the context of the viral replication machinery. However, we cannot rule out the possibility that the effect of dasatinib on translation is linked to virus-mediated changes in the cellular environment that may not be fully recapitulated in this system.

### Dasatinib and Torin 1, but not rapamycin, block eIF4E phosphorylation.

SFKs modulate a complex array of signaling pathways, including the Raf/MEK/Erk and the Akt/mTOR pathways (81–83). The Raf/MEK/Erk pathway and p38 mitogen activated protein (p38 MAP) kinases contribute to activation of eIF4E, which is a rate-limiting factor in the translation initiation complex of capped mRNAs (84). Figure S7A in the supplemental material confirms that dasatinib blocks Erk1/2 phosphorylation in NHDFs. To test whether signaling through the Raf/MEK/Erk/MNK cascade (MNK is mitogen-activated protein kinase interacting protein kinase) was responsible for the reduction in viral structural protein synthesis, NHDFs were infected with CHIKV and treated with U0126, a specific inhibitor of MEK1/2 (85). U0126 reduced the phosphorylation of Erk1/2, but the treatment failed to affect viral titers or CHIKV E2 protein levels (Fig. S7B). Similarly, the MNK inhibitor CGP 57380, which blocks eIF4E phosphorylation, did not block CHIKV replication (Fig. 2A). These results indicate that the dasatinib-mediated decrease in viral structural protein synthesis is not related to its effects on the Raf/MEK/Erk signaling pathway.

### ONNV, MAYV, and RRV are sensitive to dasatinib and Torin 1.

**FIG 9:**
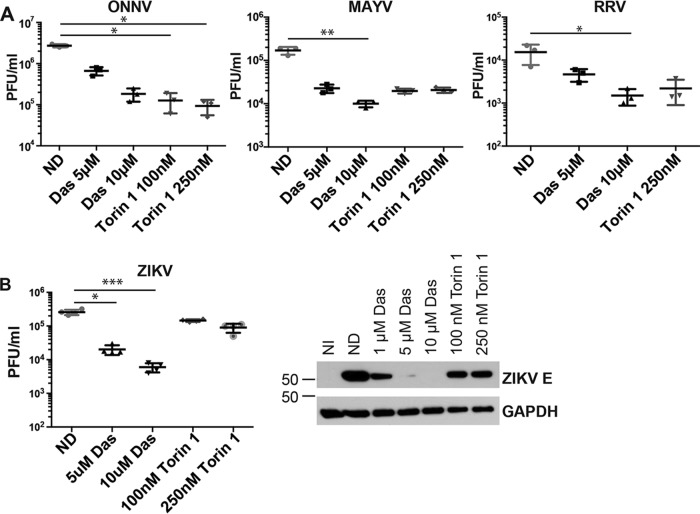
Dasatinib inhibits MAYV, ONNV, RRV, and ZIKV replication in NHDFs. (A) NHDFs were infected with ONNV, MAYV, or RRV (MOI = 3 PFU/cell) and treated with the inhibitor at 2 hpi. Supernatants were collected at 24 hpi, and titers were determined on Vero cells (*n* = 3). (B) NHDFs were infected with ZIKV (MOI = 3 PFU/cell) and treated with dasatinib at 2 hpi (*n* = 4). Supernatants were collected at 48 hpi, and titers were determined on Vero cells. Lystates from infected cells were analyzed for ZIKV E and GAPDH by Western blotting. Statistical analyses were performed on log-transformed data, data were analyzed by Dunn’s multiple-comparison test, and multiplicity-adjusted *P* values are reported (*, *P* < 0.05; **, *P* < 0.005; ***, *P* < 0.0005).

We next examined the phylogenetic breadth of viruses sensitive to dasatinib and Torin 1. NHDFs were infected with ONNV, MAYV, or RRV and treated with dasatinib or Torin 1. At 20 hpi, titers in the supernatants from infected cells were determined by serial-dilution plaque assays (Fig. 9A). Titers of ONNV, MAYV, and RRV were all significantly reduced by dasatinib and Torin 1, suggesting a common mechanism of action against a broad range of alphaviruses. To determine if a more distantly related mosquito-transmitted RNA virus was also sensitive to dasatinib and/or Torin 1, we tested the ability of these inhibitors to block the replication of the flavivirus Zika virus (ZIKV). Dasatinib treatment of ZIKV-infected NHDFs blocked viral replication; however, Torin 1 failed to block ZIKV infection (Fig. 9B). Consistent with the viral titers, ZIKV E protein levels were reduced by dasatinib but only moderately decreased by Torin 1. Together, these data indicate that CHIKV, VEEV, ONNV, RRV, and MAYV are sensitive to both dasatinib and Torin 1. However, ZIKV exhibits different sensitivities to Torin 1 and dasatinib, indicating a unique requirement for the Akt/mTOR pathway.

## DISCUSSION

**FIG 10:**
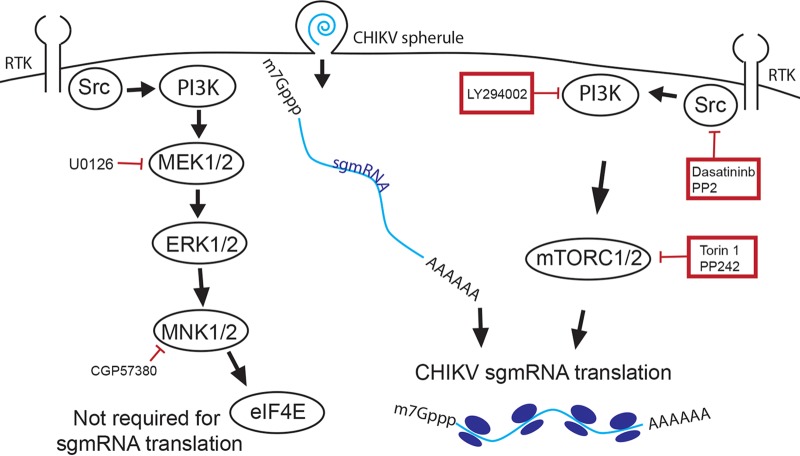
Dasatinib and Torin 1 block translation of CHIKV sgmRNA. In this study, we found that CHIKV is sensitive to the SFK inhibitor dasatinib, the PI3K inhibitor LY294002, and the mTORC1/2 inhibitors Torin 1 and PP242. We show that dasatinib and Torin 1 block CHIKV sgmRNA translation. In contrast, inhibitors of the Ras/Raf/MEK/ERK pathway did not block CHIKV sgmRNA translation.

Alphaviruses such as CHIKV, VEEV, MAYV, and ONNV are emerging pathogens that cause severe arthritis or encephalitis. Since there is no FDA-licensed antiviral therapy or vaccine, there is an urgent need for basic antiviral research to facilitate the creation of new therapeutics. In this study, we sought to understand the importance of SFK signal transduction during alphavirus infection. We found that the SFK inhibitor dasatinib and the mTORC1/2 inhibitor Torin 1 were effective in blocking virus replication at the level of structural protein synthesis (Fig. 10). Mechanism-of-action studies demonstrated that both dasatinib and Torin 1 had minimal to no effect on the formation of viral replication complexes or levels of vRNA. However, both drugs dramatically inhibited viral structural protein synthesis. Polysome profiling revealed that there were fewer CHIKV viral mRNA transcripts associated with translation-active ribosomal complexes in dasatinib-treated cells. These results demonstrate that dasatinib-mediated inhibition of alphaviruses occurs at a replication stage between viral RNA transcription and structural protein synthesis.

Dasatinib is a broadly acting SFK inhibitor that affects several cellular processes, and many of these processes may be required for effective alphavirus replication. We show that SFK inhibition blocks infection in the absence of functional interferon signaling in cells lacking IRF3 or STAT1. We also demonstrate that dasatinib, unlike Torin 1, does not induce autophagy, as indicated by LC3-II accumulation. Interestingly, inhibition of autophagy by 3-MA increased E2 accumulation, suggesting that autophagy has a negative impact on viral replication. Thus, promotion of autophagy by Torin 1 inhibition may contribute to the impairment of alphavirus translation and replication. These conclusions contradict other reports using HEK293 cells (61), but these disparities may reflect differences in cell types. Thus, these results demonstrate that dasatinib-mediated inhibition of alphavirus replication in human fibroblasts is independent of interferon signaling and autophagy processes.

We hypothesized that SFK signaling events are required for translation of viral sgmRNAs. In addition to inhibiting viral structural protein translation during infection, dasatinib treatment also blocked the translation of replicon-derived sgmRNAs. Importantly, we show that dasatinib did not inhibit translation of host mRNAs or exogenously transfected sgmRNAs but that its effects were specific to the replicon-derived sgmRNAs. These results are reminiscent of those from previous studies with SINV replicons showing that the translational behavior of transfected subgenomic mRNAs is different from that of subgenomic mRNAs derived from replicons (86, 87). SINV replicon-derived sgmRNAs were reported to be translated efficiently in the presence of the eIF4 inhibitor arsenite, and the replicon-derived sgmRNA had reduced requirements for eIF2α, eIF4A, and eIF4G (29, 77). In contrast, translation of the exogenously transfected sgmRNA was sensitive to arsenite and required eIF4G. Therefore, we found that dasatinib targets the translation processes unique to the arsenite-insensitive sgmRNA but not other mRNAs.

During alphavirus infection, translation of host mRNAs is strongly inhibited, while translation of viral sgmRNAs is very efficient (69, 88, 89). Although host translation inhibition can occur as a result of virus-induced inactivation of eIF2α by the dsRNA-sensing protein kinase R (PKR), we observed host translation shutoff in PKR^−/−^ cells, where eIF2 is still active, suggesting an alternative mechanism (69, 90). Consistent with those previous findings, we show that CHIKV induced eIF2α phosphorylation in a PKR-dependent manner. In addition, dasatinib enhanced eIF2α phosphorylation in PKR^+/+^ and PKR^−/−^ cells. eIF2α is phosphorylated by one of four kinases: heme-regulated inhibitor (HRI), PKR-like endoplasmic reticulum kinase (PERK), general control nondepressible 2 (GCN2), or PKR (91). As such, dasatinib may induce eIF2α phosphorylation through one of these kinases. One hypothesis based on our results is that the virus requires eIF2 for its replication and that dasatinib tips the balance of active versus inactive eIF2 available for the viral sgmRNA. Alternatively, the virus does not require active eIF2 for translation of its sgmRNA, and dasatinib mediates another effect on the cell that blocks the synthesis of viral structural proteins.

Previous findings regarding the translation of alphavirus sgmRNA support a model in which alphaviruses do not require activated eIF2 (28, 32, 92). SINV possesses a structural element in the 5′ end of the sgmRNA called a downstream loop (DLP), which promotes stalling of the ribosome and negates the need for an activated eIF2 (28, 31, 32). Other alphaviruses either possess similar DLP structures with different stabilities and stem lengths or have no predicted DLP-like structure (31). CHIKV, VEEV, and ONNV do not possess a predicted DLP structure and may have a different feature that serves the same function. These alphaviruses may have differential requirements for translation initiation. Conversely, MAYV has a predicted DLP structure, but it was still sensitive to dasatinib treatment. More experiments are needed to determine whether dasatinib-mediated inhibition of eIF2 is the mechanism of action for inhibiting translation of the viral sgmRNA or whether a different feature of translation is blocked.

To identify an SFK-dependent signaling pathway that modulates CHIKV replication, we utilized a panel of inhibitors specific for downstream molecules of the Ras/Raf/MEK/ERK and PI3K/Akt/mTORC1 signaling pathways. We show that treatment with U0126, an inhibitor of MEK1/2, did not affect viral protein synthesis or levels of infectious virus. This indicated that the effect of dasatinib on viral protein synthesis was mediated by another signaling event, such as the PI3K/Akt/mTOR pathway. Previous studies found that mTORC1 inhibition by rapamycin and other rapalogs could enhance CHIKV replication, presumably by enhancing the phosphorylation of eIF4E (62). Rapamycin inhibition of mTOR may result in feedback activation of Akt and increase eIF4E phosphorylation, while PI3K inhibitors and dual mTORC1-mTORC2 inhibitors decrease eIF4E phosphorylation (93–95). This feedback loop could explain the discordant effects of Torin 1 and rapamycin treatments on alphavirus replication. We found, however, that CGP 57380, an MNK1/2 inhibitor that blocks eIF4E phosphorylation, did not block CHIKV replication, suggesting that eIF4E may not be necessary for translation of the CHIKV sgmRNA.

Our data indicate that SFK signaling events play a crucial role during alphavirus replication at the step of structural protein translation but not at the level of viral RNA synthesis. Therefore, further research on these pathways will facilitate the development of novel therapeutics that target the translation step of alphavirus replication and provide insight into the signaling pathways required for efficient replication.

## MATERIALS AND METHODS

### Cells.

Normal human dermal fibroblasts (NHDFs) (ATCC PCS-201-012), Vero cells (ATCC CCL-81), Src/Yes/Fyn knockout mouse fibroblasts (68) (SYF^−/−^) (ATCC CRL-2459), and SYF^−/−^ mouse fibroblasts reconstituted with Src (SYF^+^ c-Src) (ATCC CRL-2498) were grown at 37°C in complete Dulbecco’s modified Eagle medium (DMEM; Corning) containing 5% or 10% fetal calf serum (Thermo Scientific) and supplemented with 1× penicillin-streptomycin-glutamine (Life Technologies). Aedes albopictus C6/36 cells (96) (ATCC CRL-1660) were grown at 28°C in complete DMEM. STAT1^−/−^ telomerized human fibroblasts (THF-ISRE-ΔSTAT1), IRF3^−/−^ telomerized human fibroblasts (THF-ISRE-ΔIRF3), and PKR^−/−^ telomerized human fibroblasts (THF-ISRE-ΔPKR) were generated as previously described and grown under antibiotic selection (71). Cell viability was assessed using the CellTiter-Glo luminescent cell viability assay kit (Promega) according to the manufacturer’s instructions.

### Viruses.

VEEV_TC83_ was obtained from Michael Diamond (Washington University, St. Louis, MO). ONNV, MAYV, and RRV were obtained from Robert Tesh (University of Texas Medical Branch, Galveston, TX). Studies with CHIKV used viruses derived from infectious clones of CHIKV SL15649-pMH56 (97) and CHIKV_181/25_ (98) as well as the CHIKV replicon pMH42 (99). Virus stocks were propagated in C6/36 insect cells by infection at a low multiplicity of infection (MOI). At 48 to 72 h postinfection (hpi), CHIKV, VEEV, MAYV, ONNV, and RRV stocks were produced by pelleting clarified supernatants through a 15% sucrose cushion by ultracentrifugation (25,000 rpm [76,618 × *g*] for 1.5 h in an SW 32 Ti rotor). Virus pellets were resuspended in phosphate-buffered saline and stored at −80°C. PFU for all viral stocks were determined by serial-dilution plaque assays on Vero cells using a carboxymethylcellulose overlay. At 48 hpi, cells were fixed with 3.7% formaldehyde and stained with methylene blue, and the plaques were enumerated to determine virus titers. All MOI calculations were based upon these Vero cell titers. Thus, actual productive infection levels may vary depending upon the cell type and virus strain used for each experiment.

### Kinase inhibitors.

Dasatinib, Torin 1, and rapamycin were obtained from LC Laboratories (Boston, MA). PP242 and RAD001 were obtained from Selleckchem (Houston, TX). PP2, U0126, and GSK 690693 were obtained from Sigma-Aldrich (St. Louis, MO). GSK 650394 was obtained from Santa Cruz Biotechnology, Inc. (Santa Cruz, CA). LY294002 was obtained from Cell Signaling (Danvers, MA). CGP 57380 was obtained from Abcam (Cambridge, MA).

### Kinome profiling.

Human dermal fibroblasts were infected with CHIKV_SL15649_ at an MOI equal to 5 PFU/cell. At 0, 4, 8, 12, and 18 hpi, cells were washed with PBS and then lysed in MIB-MS lysis buffer (50 mM HEPES [pH 7.5], 150 mM NaCl, 0.5% Triton X-100, 1 mM EDTA, 1 mM EGTA, 10 mM NaF, and 2.5 mM NaVO_4_ plus a protease inhibitor cocktail [Roche], phosphatase inhibitor cocktail 2 [catalog number P5726; Sigma], and phosphatase inhibitor cocktail 3 [catalog number P0044; Sigma]). Lysates were sonicated, clarified by centrifugation at 14,000 × *g*, and passaged through a 0.2-μm filter. The protein concentration in each lysate was quantified by the Bradford assay (100). MIB-MS columns composed of 4 immobilized kinase inhibitors (PP58, purvalanol B, VI16832, and UNC21474) were equilibrated in MIB-MS high-salt buffer (50 mM HEPES [pH 7.5], 1 M NaCl, 0.5% Triton X-100, 1 mM EDTA, and 1 mM EGTA) prior to the addition of clarified lysates. MIB-MS columns were washed with MIB-MS high-salt buffer, MIB-MS low-salt buffer (50 mM HEPES [pH 7.5], 150 mM NaCl, 0.5% Triton X-100, 1 mM EDTA, and 1 mM EGTA), and SDS wash buffer (50 mM HEPES [pH 7.5], 150 mM NaCl, 0.5% Triton X-100, 0.1% SDS, 1 mM EDTA, and 1 mM EGTA). Proteins were eluted from the beads by boiling for 15 min in sample buffer (200 mM Tris-HCl [pH 6.8], 0.5% SDS, 1% β-mercaptoethanol). Dithiothreitol (DTT) was then added to the samples (5 mM final concentration), and the samples were incubated at 60°C for 20 min. Iodoacetamide was added to a final concentration of 20 mM, and the samples were incubated for 30 min at room temperature in the dark. Samples were concentrated to a final volume of ∼100 μl using 10,000-molecular-weight (10K)-cutoff Amicon Ultra centrifugal concentrators, and proteins were purified by methanol-chloroform extraction. Samples were resuspended in 50 mM HEPES (pH 8.0) prior to digestion with sequencing-grade trypsin (Promega) overnight at 37°C. Residual detergent was removed by extraction with ethyl acetate, and samples were desalted with C_18_ spin columns (Pierce) according to the manufacturer’s protocol. Samples were analyzed by liquid chromatography-tandem mass spectrometry (LC-MS/MS) using an Easy nLC 1000 instrument coupled to a QExactive HF mass spectrometer (Thermo Scientific). Peptides were separated on an Easy Spray PepMap C_18_ column over a 2-h gradient of 5 to 32% mobile phase B at a 250-nl/min flow rate, where mobile phase A was 0.1% formic acid in water and mobile phase B consisted of 0.1% formic acid in acetonitrile. The QExactive HF mass spectrometer was operated in the data-dependent mode, and the 15 most-intense precursors peptides were selected for subsequent MS/MS analysis. To identify kinases, peptide data were analyzed against the current version of the human UniProt database using MaxQuant software, as previously described (57, 58). Peptide data are available in Table S1 in the supplemental material. Temporal kinome shifts and clustering were determined by k-means clustering performed in the R statistical programming language. Kinase clustering data are presented in Table S2.

### Quantitative RT-PCR analysis.

Viral RNA in infected NHDFs was quantified using real-time quantitative RT-PCR. Primers and probes include forward primer GAGGTGTGGGACTGGTTGTTG, reverse primer CAAGTTAGTGCCTGCTGAACGA, and probe AATCGTGGTGCTATGCGT for CHIKV-LR E1; forward primer CGGCGTCTACCCATTTATGT, reverse primer CCCTGTATGCTGATGCAAATTC, and probe AAACACGCAGTTGAGCGAAGCAC for CHIKV E1; forward primer AGAGACCAGTCGACGTGTTGTAC, reverse primer GTGCGCATTTTGCCTTCGTA, and probe ATCTGCACCCAAGTGTACCA for CHIKV nsP2; and forward primer TCCCGACGCCTTGTTCAC, reverse primer CGCCAAAGTCGGATGAATACA, and probe CACCGACACTTTCAGCGG for VEEV_TC83_ E2. Total RNA was prepared from infected NHDFs by the TRIzol method at 0, 2, 4, 6, 8, 12, and 24 hpi. RNA was treated with RNase-free DNase, and single-stranded cDNA was then generated using random hexamers and Superscript III RT (Invitrogen). Gene amplicons served as quantification standards (the limit of detection is approximately 10 to 100 copies). Quantitative RT-PCR results were analyzed using an ABI StepOne Plus real-time PCR system (Applied Biosystems).

### RNA analysis by Northern blotting.

NHDFs were infected with CHIKV_181/25_, and total RNA was collected at 12 hpi by the TRIzol method. RNA was electrophoretically separated on a formaldehyde agarose gel and transferred onto a Hybond-N^+^ positively charged nylon membrane (Amersham). CHIKV RNA was detected using an E2-6K-E1-specific digoxigenin (DIG) probe (Roche) constructed by PCR using forward primer 5′-CGCAGTTATCTACAAACGGTA-3′ and reverse primer 5′-TTTACTCTCAGGTGTGCGA-3′. Human β-actin RNA was detected using a DIG probe constructed using forward primer 5′-ACCCTGAAGTACCCCATCGA-3′ and reverse primer 5′-CGGACTCGTCATACTCCTGC-3′. Detection was performed using the DIG-High Prime DNA labeling and detection starter kit II (Roche). DIG-labeled membranes were incubated with a CSPD [disodium 3-(4-methoxyspiro {1,2-dioxetane-3,2′-(5″-chloro)tricyclo (3.3.1.1)decan}-4-yl)phenyl phosphate] alkaline phosphatase chemiluminescent substrate and visualized on CL-XPosure film (Thermo).

### *In vitro* mRNA synthesis and mRNA transfections.

A CHIKV subgenomic mRNA construct expressing an eGFP-HA-tagged fusion protein (sg-HA) was generated as follows: the CHIKV 5′ sgUTR was amplified from pMH56 using forward primer 5′-ATATAAGCTTCGTCATAACTTTGTACGGCGG-3′ and reverse primer (5′-CTCCTCGCCCTTGCTCACCATTGTAGCTGATTAGTGTTTAG-3′. The eGFP-HA fragment was amplified using forward primer 5′-CTAAACACTAATCAGCTACAATGGTGAGCAAGGGCGAGGAG-3′ and reverse primer 5′-ATATGGATCCTTAGGCGTAGTCGGGCACATCGTACGGGTACTTGTACAGCTCGTCCATGC-3′. The sg-HA fusion gene was synthesized by overlapping PCR and cloned into the pSP64 poly(A) vector. For vRNA synthesis, the CHIKV_181/25_ cDNA clone and pMH42 were first linearized with NotI (NEB), or pSP64 was digested with EcoRI (NEB). Capped and polyadenylated mRNA transcripts were generated from the linearized plasmids using the mMessage mMachine SP6 transcription kit (Thermo Fisher). Samples were digested with Turbo DNase I, and mRNAs were purified using the RNeasy Plus minikit (Qiagen). mRNAs were transfected into fibroblasts using Lipofectamine MessengerMAX (Invitrogen) according to the manufacturer’s instructions.

### Protein analysis.

Infected cells were washed with ice-cold PBS and lysed with cell lysis buffer (Cell Signaling) supplemented with 1 mM phenylmethylsulfonyl fluoride (PMSF; Fisher) on ice for 15 min. Lysates were collected and centrifuged at 4°C for 10 min at 13,200 rpm. The supernatant was boiled in Laemmli’s sample buffer and analyzed by SDS-PAGE using 4 to 12% Bis-Tris polyacrylamide gels (Life Technologies). Proteins were transferred onto polyvinylidene difluoride (PVDF) membranes by semidry transfer, and the blots were blocked with 5% milk or 5% bovine serum albumin (BSA; Fisher) in Tris-buffered saline with 0.1% Tween (TBST). Immunoblots were incubated with primary antibodies directed against CHIKV E2 (obtained from Michael Diamond), nsP3, a β-actin–horseradish peroxidase (HRP) conjugate (13E5), GAPDH (glyceraldehyde-3-phosphate dehydrogenase)-HRP (14C10), pS6 ribosomal protein (Ser235/236), S6 (5G10), the p-Src family (Y416), p44/42 Erk1/2 (Thr202/Tyr204), p-4EBP1 (236B4), 4EBP1, p-eIF4E (S209), eIF4E (C46H6), and ATF4 (D4B8) (Cell Signaling); LC3B (Sigma-Aldrich); and anti-Erk 1 (C-16), anti-LC3B, c-Src (Src2), and PKR (K-17) (Santa Cruz). Antibodies directed against the VEEV envelope glycoprotein (gp) and VEEV capsid were obtained through BEI Resources, NIAID, NIH, as part of the Human Microbiome project. Primary antibodies were diluted 1:1,000 in 5% milk in TBST or 5% BSA and incubated for 1 h at room temperature or overnight at 4°C. The blots were washed with 10 volumes of TBST and then incubated for 30 min with horseradish peroxidase-linked secondary antibodies diluted 1:10,000 in milk-TBST or BSA-TBST. After washing, the positive bands were detected after the addition of chemiluminescence reagents and visualized on CL-XPosure film (Thermo).

### PathScan RTK antibody array.

PathScan RTK signaling phosphoantibody array analysis (Cell Signaling) was performed according to the manufacturer’s protocol. Briefly, lysates from NHDFs infected with CHIKV_SL15649_ at an MOI of 1 PFU/cell were collected at 0, 1, 2, 4, 6, 8, and 24 hpi. Protein concentrations were normalized, and the samples were incubated on an RTK antibody array chip for 2 h. The plates were washed and incubated with a detection antibody cocktail for 1 h. The plates were washed and incubated with a DyLight 680-linked streptavidin secondary antibody, after which they were washed, dried, and scanned using an Odyssey infrared imaging system (Li-Cor). Spot intensities were quantified with Odyssey quantification software and are reported relative to values for the uninfected samples.

### Immunofluorescence.

Cells were plated onto 4-well Lab-Tek chambered cover glass slides (Fisher) and infected as indicated. Cells were fixed with 4% paraformaldehyde for 15 min and washed with PBS. Cells were incubated for 1 h with permeabilization buffer containing 0.2% saponin and 1% BSA. Cells were incubated overnight, as indicated, with anti-CHIKV E2, anti-VEEV gp, or anti-dsRNA (J2; Scicons) diluted 1:1,000 in the presence of saponin and BSA. Cells were washed and incubated with goat anti-mouse Alexa Fluor 488 (Abcam), DAPI, and/or anti-mouse Texas Red-X phalloidin for 1 h. Cells were washed and visualized using a confocal fluorescence microscope.

### Polysome analysis.

NHDFs were mock infected or infected with CHIKV_181/25_ (MOI = 3 PFU/cell). At 2 hpi, medium was removed, and cells were washed twice with PBS. The medium was replaced with or without 10 μM dasatinib. At 12 hpi, 0.1 mg/ml cycloheximide (Sigma-Aldrich) was added to the medium, and cells were incubated for 10 min at 37°C. Following incubation, cells were washed twice with ice-cold PBS containing 0.1 mg/ml cycloheximide. The cells were scraped, pelleted for 10 min at 1,130 × *g*, and frozen at −80°C. Samples were resuspended in polysome buffer (20 mM Tris-HCl [pH 7.4], 140 mM KCl, 5 mM MgCl_2_) containing 0.1% Triton X-100, 10 mM DTT, and 100 μM CHX. Cells were disrupted by five passes through a 27-gauge needle, and nuclei were removed by centrifugation for 5 min at 1,150 × *g*. The resulting cytoplasmic lysates were layered onto 10 to 50% linear sucrose gradients prepared in polysome buffer and centrifuged at 35,000 × *g* for 2 h at 4°C with no brake. The gradients were fractionated with continuous absorbance monitoring at an OD_254_ using a gradient fractionation system (Brandel). RNA was isolated from each fraction and quantified by qRT-PCR using the absolute quantification method. Translation efficiency was calculated as the sum of CHIKV RNA copies in the polysome-containing fractions (fractions 9 to 15) divided by the sum of CHIKV RNA copies in the fractions containing ribosomal subunits and monosomes (fractions 4 to 7) (*n* = 2).

## Supplementary Material

Supplemental file 1

Supplemental file 2
