# Supplementary material for: Src Family Kinase Inhibitors Block Translation of Alphavirus Subgenomic mRNAs
Source: Antimicrob Agents Chemother. 2019 Mar 27;63(4):e02325-18. doi: 10.1128/AAC.02325-18 (PMC6496153; doi:10.1128/AAC.02325-18)
Supplement: Supplemental file 1 [file AAC.02325-18-s0001.pdf]

Supplmental Figures and Tables

Src Family Kinase Inhibitors Block Translation of Alphavirus Subgenomic mRNAs

Rebecca Broeckel[1], Sanjay Sarkar[3], Nicholas A. May[4], Jennifer Totonchy[1,#], Craig N. Kreklywich[1], Patricia Smith[1], Lee Graves[4], Victor R. DeFilippis[1,2], Mark Heise[3,5], Thomas E. Morrison[6], Nathaniel Moorman[5], and Daniel N. Streblow[1,2,*]

Vaccine and Gene Therapy Institute, Oregon Health and Science University, Beaverton, Oregon[1]; Division of Pathobiology and Immunology, Oregon National Primate Research Center, Beaverton, Oregon[2]; Department of Genetics, The University of North Carolina at Chapel Hill, Chapel Hill, North Carolina[3]; Department of Pharmacology, The University of North Carolina[4], Department of Microbiology and Immunology, The University of North Carolina[5], Department of Immunology and Microbiology, University of Colorado School of Medicine, Aurora, Colorado[6]

Running Head: Inhibiting alphavirus protein synthesis

*Address correspondence to Daniel N. Streblow, streblow@ohsu.edu

#Present address: Department of Biomedical and Pharmaceutical Sciences, Chapman University School of Pharmacy, Irvine, California.

Supplemental Table 2. K means clustering of cellular kinome following CHIKV infection.

Cluster 1			Cluster 2	Cluster 3
TK2	CSK	CAMK2D	AURKA	BRAF;ARAF;RAF1
PIK3C2A	MTOR	DYRK1A	DAPK3	NME1
STK25	ABL2	PRKG1	INSR	EIF2AK2
NME4	MAPK8	PRKD1	FGFR1	MAP2K4
PDXK	MAPK9	EPHA7	PDGFRA	MAPK10
GAK	MAP2K3	RPS6KA1	PFKL	MAP4K2
RIPK2	PIP4K2A	MYLK	JAK1	MAPK7
PIP5K1C	CSNK1A1;CSNK1A1L	MAPK11	BMPR1A	CAMK2B
STK16	CSNK1D	MAPK14	ACVR1B	STK11
STK10	CSNK1E	MAP3K11	TGFBR1	TAOK1
PRKD3	GSK3A	AAK1	TGFBR2	STRADA
MAP4K4	GSK3B	ULK3	PI4KA	PI4K2B
PAK4	CDK7	MARK2	TEC	SRPK1
ABL1	CDK9	ADCK1	CLK3	TAOK3
EGFR	RPS6KA3	VRK2	EPHA4	IRAK4
PGK1;PGK2	MAP2K6	MAP4K3	ACVR1	NLK
FYN	LIMK1	MINK1	TYRO3	STK24
CDK1	EPHB3	PIK3C3	DDR1	
FES	EPHB4	PIP4K2C	PRPF4B	
YES1	CSNK2A1;CSNK2A3	NEK9	BMPR2	
LYN	PIP4K2B	MARK4	CDK13	
IGF1R	SRPK2	TP53RK	MELK	
PDGFRB	CSNK1G2	PRKD2	DDR2	
CDK4	PRKDC	SIK2	NEK7;NEK6	
SRC	CDK6	SLK	AURKB	
FER	CDK5	FN3KRP	NEK1	
PRKCA	CDK16	BMP2K	PKMYT1	
PRKACA	CDK17	ZAK	RIOK2	
CSNK2A2	MAP2K1	CDK12	CLK4	
NME2;NME2P1	TEK	TBK1	CSNK1G1	
PRKACB	PTK2	TNIK	ICK	
CDK2	TNK2	TAOK2	PIKFYVE	
MAPK3	DMPK	SIK3		
MARK3	STK4	MAP3K2		
DCK	PRKAA1	MAP4K5		
MAPK1	MAP2K5	CDC42BPB		
EPHA2	STK3	CSNK1G3		
EPHB2	ATM	MAP3K4		
GK;GK3P	ILK			
MAP2K2	CAMK2G			

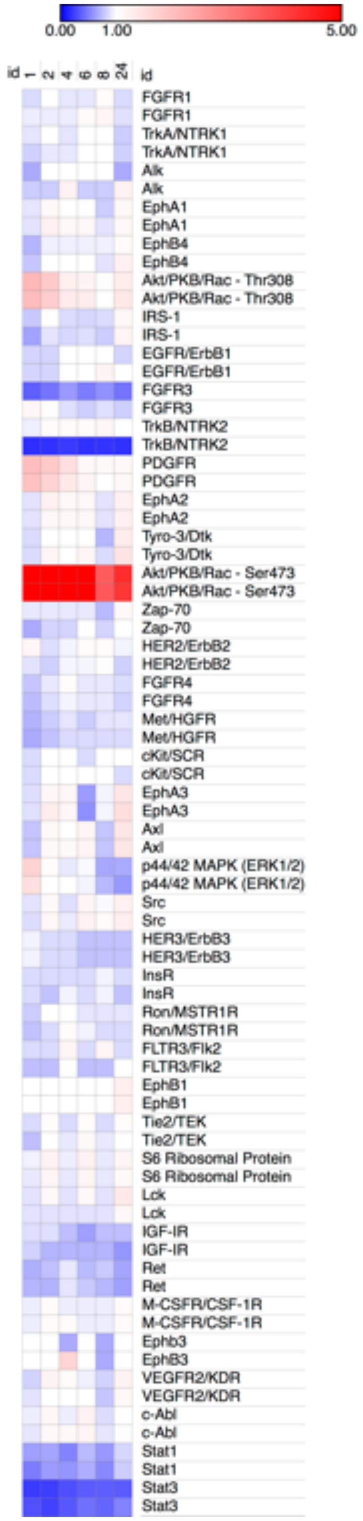

Supplemental Figure 1. Receptor tyrosine kinase antibody array chip reveals early and late phosphorylation patterns in CHIKV-infected human fibroblasts. NHDFs were infected with CHIKV SL15649 MOI = 1, and lysates were collected at 0, 1, 2, 4, 6, 8, 24 hpi. Lysates were incubated on a receptor tyrosine kinase antibody array chip (Cell Signaling), and phospho-protein levels were quantified. Levels of the phospho-proteins are reported relative to uninfected 0 hpi samples.

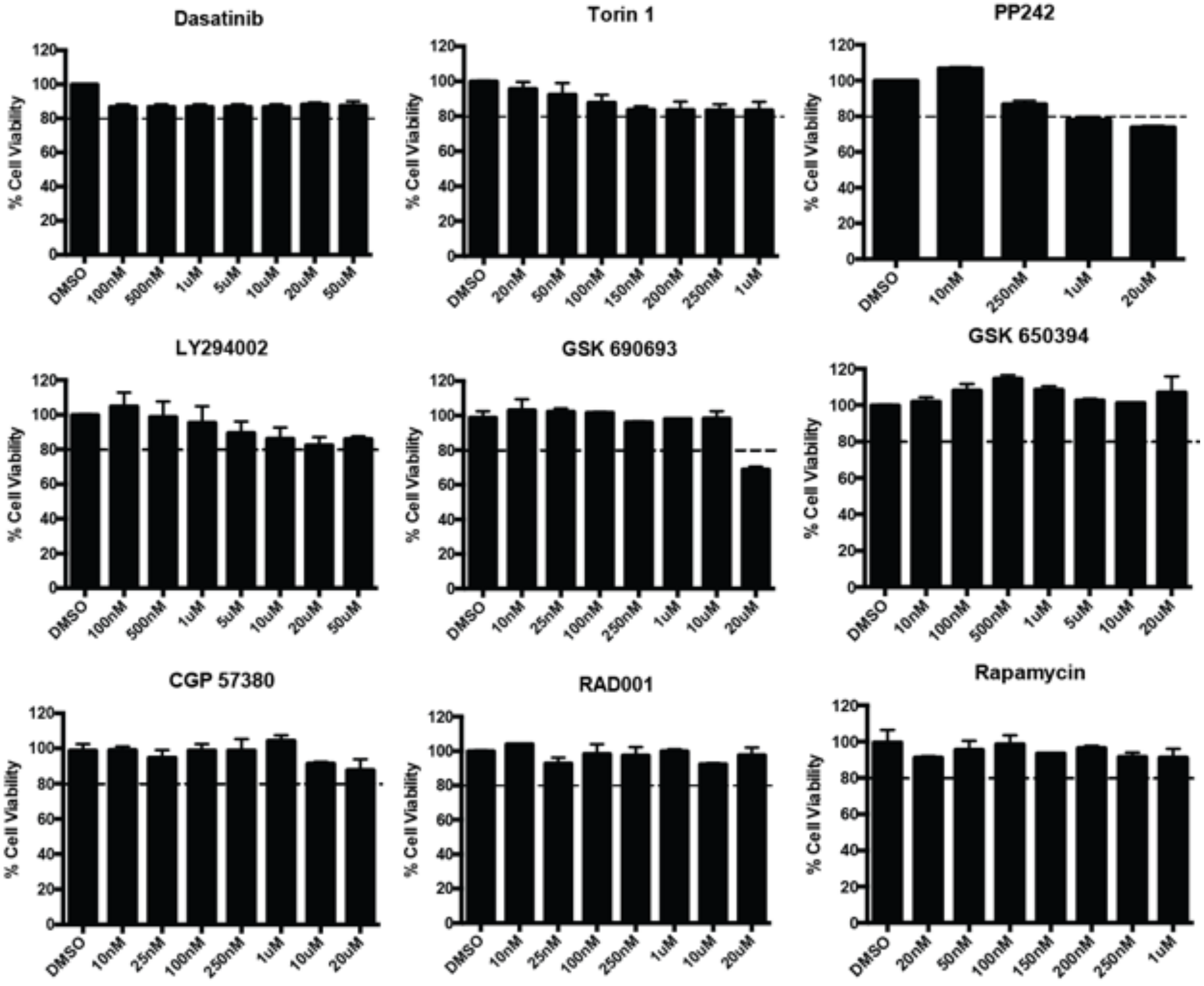

Supplemental Figure 2. Cell viability in NHDFs after treatment with different inhibitors. Cells were treated with the indicated concentrations of inhibitors. At 24 h post treatment, cell viability was measured with the CellTiter-Glo Luminsecent Cell Viability Assay kit. The dotted line marks 80% cell viability, which was the viability cutoff for our assay.

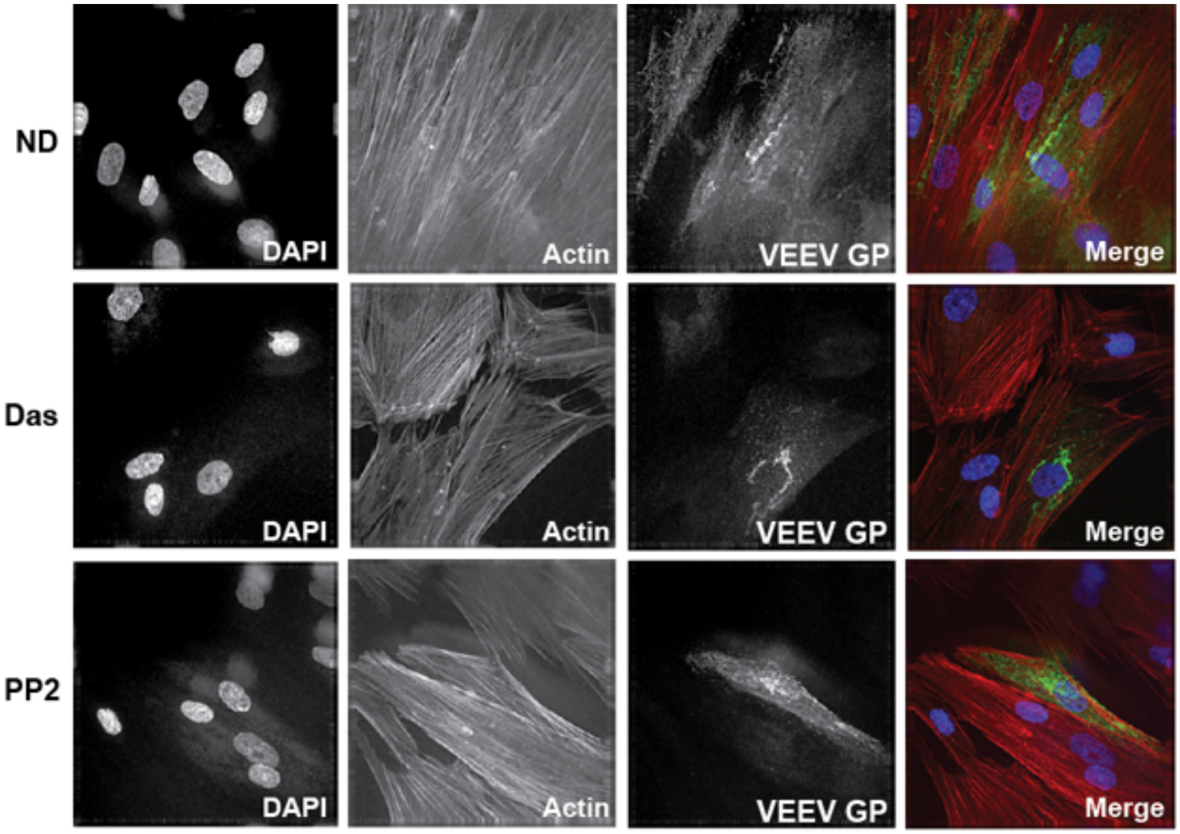

Supplemental Figure 3. Dasatinib and PP2 block viral spread from infected cells. Cells were infected with VEEV (MOI = 1 PFU/cell). At 2 hpi, cells were washed with PBS and treated with 10 μM Dasatinib or 10 μM PP2. At 24 hpi, cells were fixed, permeabilized, and stained for VEEV glycoproteins (green) and counterstained with DAPI (blue) and Phalloidin (red) to highlight host DNA and Actin filaments.

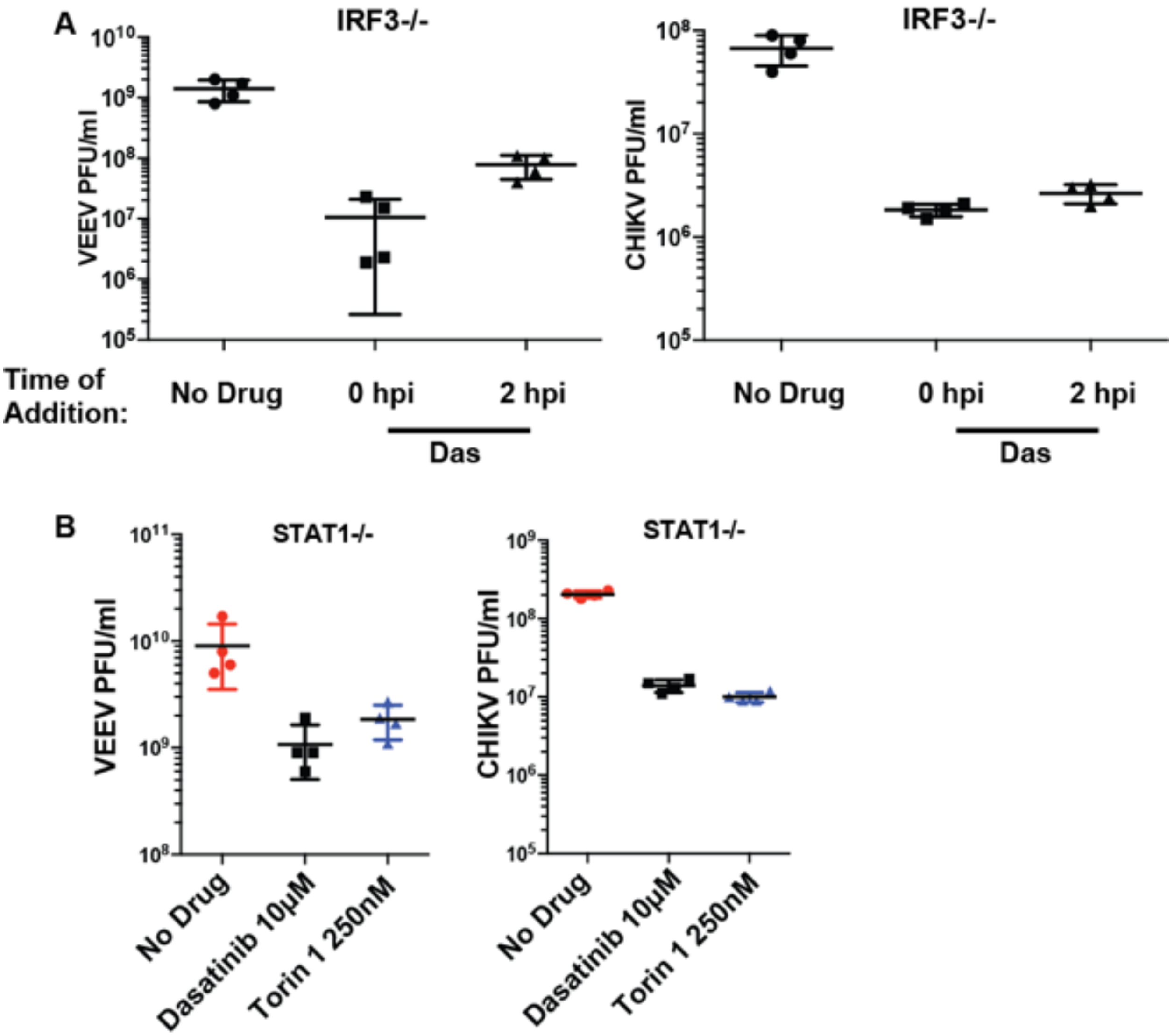

Supplemental Figure 4. Dasatinib inhibits CHIKV and VEEV replication in IRF3$^{-/-}$ and STAT1$^{-/-}$ fibroblasts. (A) IRF3$^{-/-}$ telomerized human fibroblasts were infected with CHIKV and VEEV MOI = 1 and treated with dasatinib at 0 or 2 hpi. Supernatants were taken at 24hpi from infected cells and titered on Veros by limiting dilution. (**B)** STAT1$^{-/-}$ telomerized human fibroblasts were infected with CHIKV, VEEV, or SINV (MOI = 1 PFU/cell) and treated with inhibitor at 2 hpi. Supernatants were collected at 24 hpi and titered on Veros.

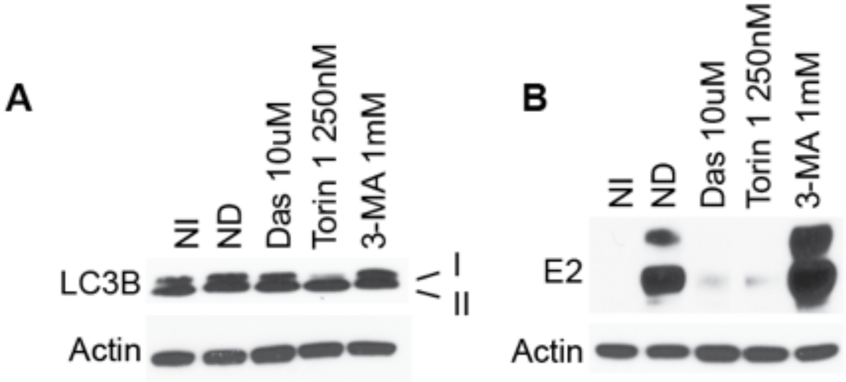

Supplemental Figure 5. Dasatinib and Torin 1 differentially affect autophagy. NHDFs were infected with CHIKV (MOI = 3 PFU/cell) or left uninfected (NI). At 2 hpi, cells were washed with PBS, and media containing drug was added to the cells as indicated. At 7 hpi, lysates were analyzed for (**A**) LC3 or (**B**) E2.

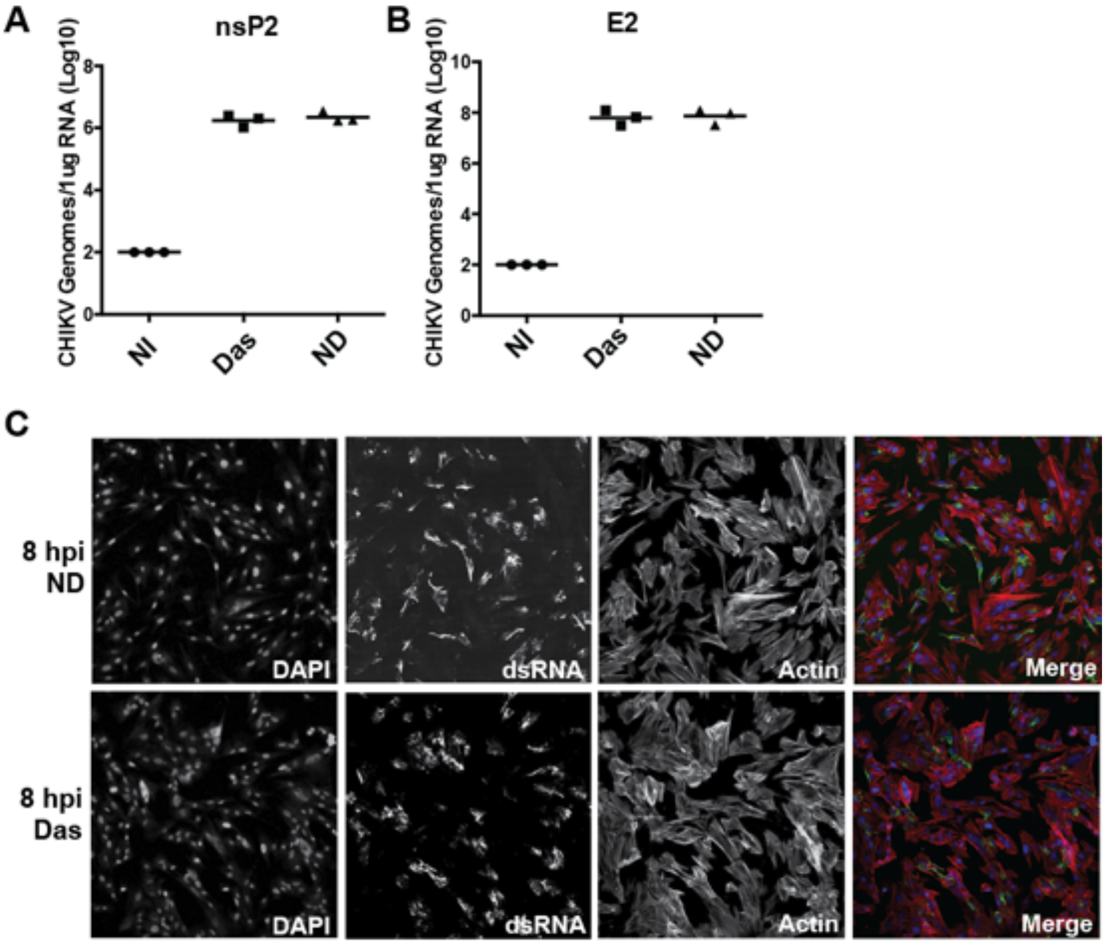

Supplemental Figure 6. Dasatinib does not affect RNA levels and dsRNA complexes. NHDFs were infected with CHIKV$_{181\text{-}25}$ (MOI = 3 PFU/cell). At 2 hpi, cells were washed with PBS, and media with no inhibitor or 10 μM dasatinib was added back to the cells. At 12 hpi, cells were washed extensively in PBS, and cell lysates were scraped in Trizol. CHIKV nsP2 (**A**) and E2 (**B**) RNA levels were measured in cell lysates. (**C**) NHDFs were infected with CHIKV$_{181\text{-}25}$ (MOI = 25 PFU/cell), and cells were treated with dasatinib at 2 hpi or untreated. At 8 hpi, cells were fixed and analyzed for levels of dsRNA (green) and counterstained with DAPI (blue) and Phalloidin (red) (NI = uninfected, ND = no drug).

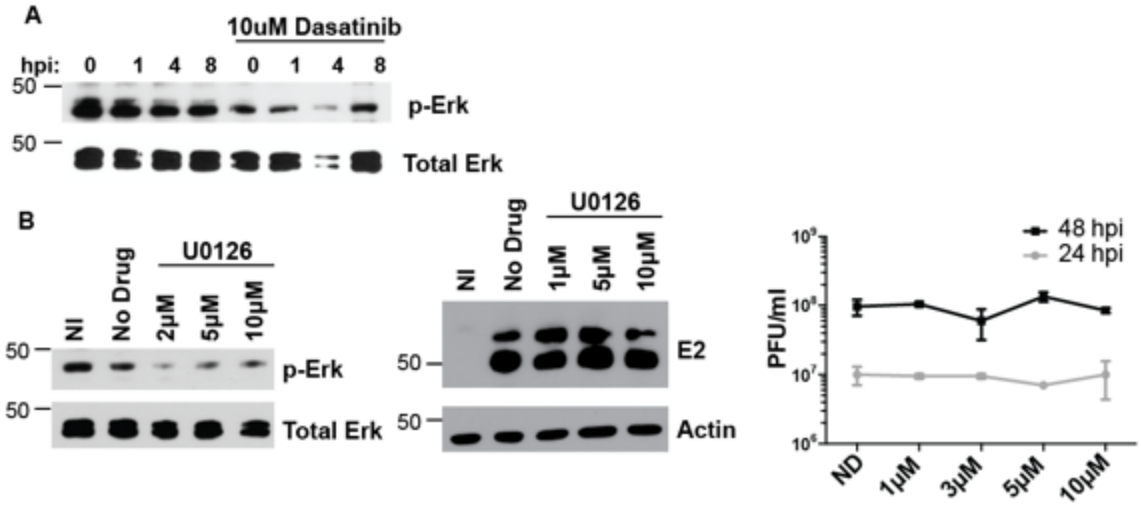

Supplemental Figure 7. Erk inhibitor U0126 does not block CHIKV infection. (**A**) NHDFs were treated with dasatinib 30 minutes prior to infection and infected with CHIKV (MOI = 1 PFU/cell). Phosphorylation of Erk and total Erk protein levels were detected by western blotting. (**B)** NHDFs were treated with U0126 at 2 hpi, and cell lyasates were analyzed at 24 hpi for p-Erk and total Erk (left panel), CHIKV E2 and Actin (center panel) by western blotting. (Right panel) NHDFs were infected with CHIKV (MOI = 1 PFU/cell), treated with U0126 at 2 hpi, and supernatants were collected at 24 hpi and 48 hpi and titered on Vero cells.
